# A101 BLASTIC PLASMACYTOID DENDRITIC CELL NEOPLASM OCCURRING POST-LIVER TRANSPLANT FOR PSC WITH CLINICAL REMISSION OF ULCERATIVE COLITIS AFTER STEM CELL TRANSPLANTATION

**DOI:** 10.1093/jcag/gwab049.100

**Published:** 2022-02-21

**Authors:** M A Donaldson, D Akhtar, J Buttar, B Salh

**Affiliations:** 1 The University of British Columbia Department of Medicine, Vancouver, BC, Canada; 2 The University of British Columbia Faculty of Medicine, Vancouver, BC, Canada

## Abstract

**Background:**

Primary sclerosing cholangitis (PSC) is a rare autoimmune fibroinflammatory disease that is associated with inflammatory bowel disease (IBD) and can progress to end-stage liver disease (ESLD) requiring liver transplantation (LT). Blastic plasmacytoid dendritic cell neoplasm (BPDCN) is a highly aggressive and rare hematologic malignancy (HM) that usually affects elderly males. High-dose chemotherapy followed by allogenic stem cell transplantation (SCT) offers the best chance of long-term remission in BPDCN. While it is well described that there is an increased risk of de-novo cancer development after solid organ transplantation (SOT), these are less commonly HMs. There are currently no documented cases of BPDCN in post-SOT patients in the literature.

**Aims:**

To describe one of the first cases of BPDCN in a patient post-SOT, and subsequent clinical resolution of UC post SCT.

**Methods:**

Case Report

**Results:**

A 19-year-old previously healthy male initially presented with cholangitis and was diagnosed with PSC. Two years after the diagnosis of PSC, he progressed to ESLD and underwent live donor LT in India. He subsequently presented with episodes of bloody diarrhea and a colonoscopy then revealed diffuse mucosal edema, erosions, and spontaneous bleeding, with scattered inflammatory polyps throughout the colon. UC was diagnosed and Infliximab therapy was started. He later presented with a UC flare and Vedolizumab therapy was initiated. A liver biopsy showed recurrent sclerosing cholangitis and ultimately the patient required a second liver transplant. After the second LT he developed weight loss, arthralgias, and progressive splenomegaly following which BPCDN was diagnosed. His Vedolizumab was subsequently stopped. He received induction chemotherapy followed by a single antigen mismatched allogenic SCT. Whilst he had numerous complications associated with his chemotherapy induction, his UC appeared to be in clinical remission. No repeat colonoscopies were completed, however, on follow-up assessments, he denied abdominal pain and reported having formed, non-bloody stools. Unfortunately, he suffered an aggressive relapse of his BPCDN, and he was palliated.

**Conclusions:**

The development of de-novo cancers after LT is common, and patients with PSC are at particularly high risk. PSC-LT patients have also been found to have the highest rate of hematological malignancies post LT; however, these are most commonly post-transplant lymphoproliferative disorder. Additionally, there have been a few reports of complete resolution of IBD after allogenic mismatched SCT. Here we describe one of the first cases of BPCDN post-LT in a PSC-UC patient and display another example of UC clinical remission post allogenic SCT. These findings emphasize that close follow-up of these patients after LT is imperative.

**Funding Agencies:**

None

